# Marine-Derived Natural Products as ATP-Competitive mTOR Kinase Inhibitors for Cancer Therapeutics

**DOI:** 10.3390/ph14030282

**Published:** 2021-03-21

**Authors:** Shraddha Parate, Vikas Kumar, Gihwan Lee, Shailima Rampogu, Jong Chan Hong, Keun Woo Lee

**Affiliations:** 1 Division of Applied Life Science, Plant Molecular Biology and Biotechnology Research Center (PMBBRC), Gyeongsang National University (GNU), 501 Jinju-daero, Jinju 52828, Korea; parateshraddha@gmail.com (S.P.); pika890131@gmail.com (G.L.); 2 Division of Life Sciences, Department of Bio & Medical Big Data (BK21 Program), Research Institute of Natural Science (RINS), Gyeongsang National University (GNU), 501 Jinju-daero, Jinju 52828, Korea; vikaspathania777@gmail.com (V.K.); shailima.rampogu@gmail.com (S.R.)

**Keywords:** mTOR kinase, marine natural products, ATP-competitive inhibitors, structure-based pharmacophore modeling, virtual screening, molecular docking, molecular dynamics simulations, binding free energy, in silico ADMET

## Abstract

The mammalian target of rapamycin (mTOR) is a serine/threonine kinase portraying a quintessential role in cellular proliferation and survival. Aberrations in the mTOR signaling pathway have been reported in numerous cancers including thyroid, lung, gastric and ovarian cancer, thus making it a therapeutic target. To attain this objective, an in silico investigation was designed, employing a pharmacophore modeling approach. A structure-based pharmacophore (SBP) model exploiting the key features of a selective mTOR inhibitor, Torkinib directed at the ATP-binding pocket was generated. A Marine Natural Products (MNP) library was screened using SBP model as a query. The retrieved compounds after consequent drug-likeness filtration were subjected to molecular docking with mTOR, thus revealing four MNPs with better scores than Torkinib. Successive refinement via molecular dynamics simulations demonstrated that the hits formed crucial interactions with key residues of the pocket. Furthermore, the four identified hits exhibited good binding free energy scores through MM-PBSA calculations and the subsequent in silico toxicity assessments displayed three hits deemed essentially non-carcinogenic and non-mutagenic. The hits presented in this investigation could act as potent ATP-competitive mTOR inhibitors, representing a platform for the future discovery of drugs from marine natural origin.

## 1. Introduction

The growth factors, nutrients and energy levels in cells are key determining factors of cellular growth and proliferation [1]. Dysregulation of the phosphoinositide 3-kinase (PI3K)/AKT/mammalian target of rapamycin (mTOR) axis has been heavily implicated in tumorigenesis and the progression of numerous cancers, including lung, thyroid, ovarian and gastric cancer [2,3,4,5]. Therefore, targeting mTOR signaling represents an attractive therapy in cancer. mTOR is a serine/threonine kinase (UniProt ID: P42345), first discovered in budding yeast Saccharomyces cerevisiae [6] and functions as a cardinal regulator of cell growth, proliferation, metabolism, energy homeostasis, angiogenesis and survival [7,8]. In mammalian cells, mTOR exists in two evolutionarily conserved complexes: mTORC1, which regulates protein synthesis through the phosphorylation of p70S6K1, and 4E-BP1 and mTORC2, which regulate cell survival and proliferation through the phosphorylation of AKT/PKB [9,10]. The mTORC1 comprises regulatory-associated protein of mTOR (RAPTOR), lethal with SEC13 protein 8 (LST8), proline-rich substrate of 40 kDa (PRAS40) and domain-containing mTOR-interacting protein (DEPTOR), while mTORC2 consists of rapamycin-insensitive companion of mTOR (RICTOR), LST8, stress-activated protein kinase interacting protein 1 (SIN1), DEPTOR and protein observed with RICTOR (PROTOR) [6]. Deregulation of mTORC1/ mTORC2 both upstream and downstream is implicated in various cancers, including breast, ovarian, prostate and lung cancer [11].

Currently, numerous pharmacological possibilities have been developed to inhibit mTOR, resulting in three generations of mTOR inhibitors. Rapamycin and its analogs (rapalogs) belong to the first generation of mTORC1 inhibitors, approved for different cancer treatments [12]. In spite of being approved, these inhibitors cause the stabilization of the disease and not tumor regression, thereby behaving as cytostatic and not as cytotoxic. Additionally, their continued use results in copious adverse effects causing suppression of the immune system, reduction in male fertility and hematological toxicity [13]. The second generation of mTOR inhibitors act as ATP-competitive inhibitors of the catalytic kinase domain controlling the activity of both mTORC1 and mTORC2 [14]. Various resistant mutations interfering with mTOR drug binding are observed in rapalogs as well as ATP-competitive kinase domain inhibitors. The third generation of mTOR inhibitors consist of bivalent drugs binding simultaneously to the regulator and mTOR catalytic sites thereby supplementing to eliminate the resistant mutations [15,16]. In recent years, several second generation of mTOR inhibitors were identified as selective ATP-competitive kinase domain inhibitors including INK-128, OSI-027 and CC-223. Moreover, dual mTOR/PI3K inhibitors, including BEZ235, PF-04691502 and GSK2126458, were also discovered as effective inhibitors. Nevertheless, these inhibitors exhibit detrimental effects including thrombocytopenia, depression, weight loss, skin rash and mucositis [17,18]. Hence, there is an emergent need to discover potent mTOR kinase domain inhibitors as therapeutic candidates for cancerous ailments.

Natural products are predominant sources of active ingredients in medicine, demonstrating varying structural diversity and exhibiting greater potential druggable pharmacophores than synthetic molecules [19,20]. Moreover, lead compounds of natural origin have proven to show significant inhibitory activities on mTOR kinase domain in the past and this provided a structural basis for identifying natural products as potent mTOR inhibitors [21,22,23]. Natural products from marine resources have recently shown therapeutic potential in the development of anti-cancer drugs [24,25,26,27,28,29,30]. It is noteworthy to mention that 7 compounds from marine organisms have been approved as pharmaceuticals for marketing, 23 compounds are in various phases of clinical trials and about 1000 compounds are undergoing preclinical studies [25]. Four marine compounds, namely cytarabine (Cytosars), trabectedin (Yondeliss), eribulin mesylate (Halavens) and the conjugated antibody brentuximab vedotin (Acentriss), are currently utilized as anti-cancer therapeutics [25]. Marine natural extracts have also exhibited significant anti-cancer effects in recent years [24,31,32,33,34]. 

The aforementioned perspectives prompted us to identify natural compounds from marine resources as potential therapeutics targeted to mTOR for treatment in cancer. In accordance with it, we have carried out an in silico study to identify mTOR inhibitors via structure-based pharmacophore modeling approach. We have used pharmacophore-based virtual screening followed by molecular docking to select candidates from a Marine Natural Product (MNP) library. Subsequently, the drug-like marine compounds showing the best docking scores and molecular interactions with the kinase domain of mTOR were further refined by molecular dynamics (MD) simulations and Molecular Mechanics Poisson–Boltzmann Surface Area (MM-PBSA) analysis. The compounds demonstrating good binding affinity scores, as revealed by MM-PBSA were confirmed as final hits and reported in this study as potential ATP-competitive mTOR kinase domain inhibitors for cancer therapeutic treatment.

## 2. Results

### 2.1. Structure-Based Pharmacophore Model

**Figure 1:**
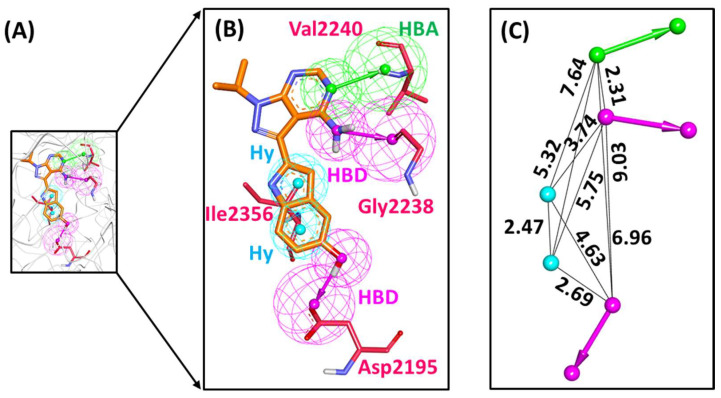
Structure-based pharmacophore model- *Pharmacophore_01*. (**A**) Pharmacophore model generated at the catalytic site of mTOR with co-crystallized ligand, PP242. (**B**) Pharmacophore features mapped with the key residues of the binding pocket. (**C**) Interfeature distance between the mapped pharmacophore features. HBA (hydrogen bond acceptor); HBD (hydrogen bond donor) and Hy (hydrophobic).

A structure-based pharmacophore was generated from the crystallographic structure of mTOR kinase complexed with PP242 inhibitor, in which the key features of PP242 binding with mTOR were exploited. Accordingly, a total of six pharmacophore models were generated with hydrogen bond donor (HBD), hydrogen bond acceptor (HBA) and hydrophobic (Hy) as common indispensable features (Table 1). The *Pharmacophore_01* with five features (1 HBA, 2 HBD and 2 Hy) and with the highest selectivity score of 9.2973 was observed to be the best pharmacophore model among the six generated pharmacophores and hence selected for further analysis. Upon meticulous examination, *Pharmacophore_01* displayed requisite features complementing the key residues for binding—Asp2195, Gly2238, Val2240 and Ile2356. The HBA feature complements with the essential residue Val2240, where PP242 is responsible for forming a hydrogen bond. The pyrazolopyrimidine scaffold of PP242 also maps on to the two HBD features by hydrogen bonding with two vital residues—Asp2195 and Gly2238. A previously published study suggests HBA and HBD as crucial features required for mTOR inhibition [21]. Moreover, the two Hy features complement the PP242 binding with Ile2356 residue via π-alkyl interactions. The Hy pharmacophoric feature was also reported as an essential feature in an earlier study on nanomolar mTOR inhibitors [35]. Therefore, *Pharmacophore_01* was escalated for further analysis (Figure 1). 

### 2.2. Decoy Set Validation of the Structure-Based Pharmacophore Model

The selected model *Pharmacophore_01* was evaluated for its efficiency in retrieving active mTOR compounds from a given database of active and inactive molecules. This validation was prompted by screening an external database (D) of 300 compounds, with 50 active compounds (A). With 61 hits retrieved from the database (Ht), active compounds obtained were 49 (Ha). The goodness of fit (GF) score was calculated as 0.80, thereby confirming that *Pharmacophore_01* can predict active compounds from a given dataset reasonably well (Table 2).

### 2.3. Drug-Like Marine Compounds Retrieved by Virtual Screening

**Figure 2:**
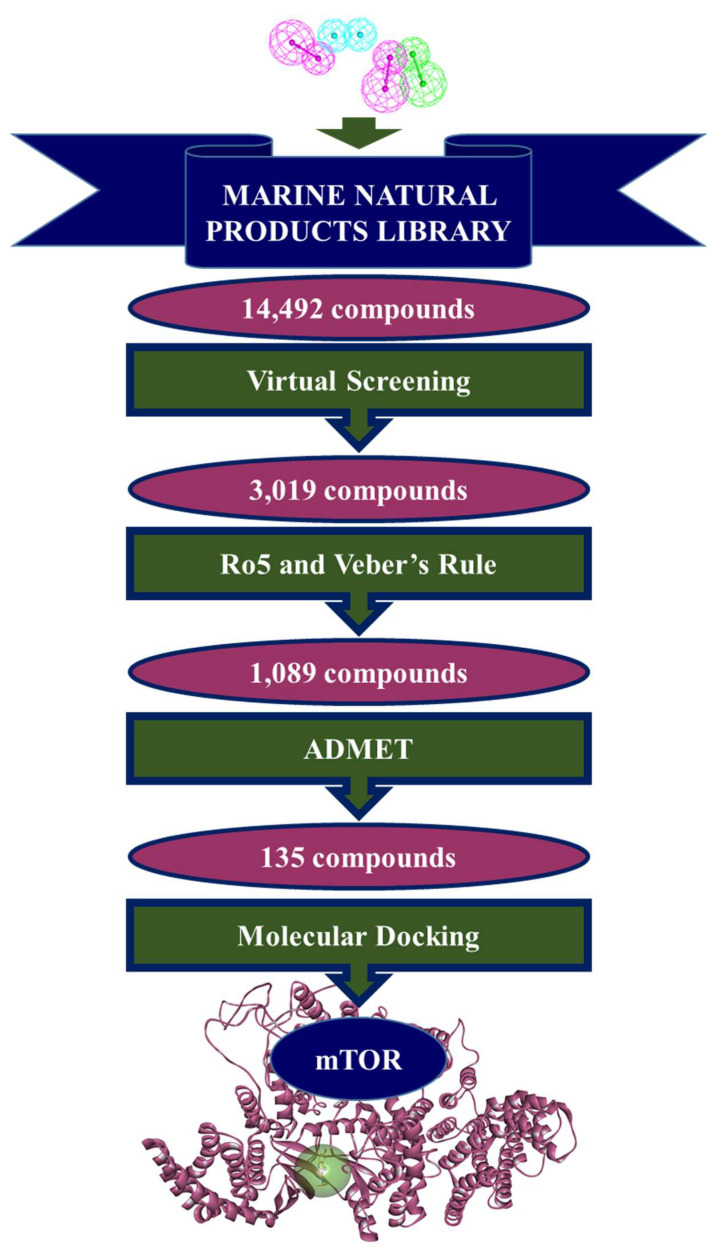
Illustration of the stages involved in the retrieval of potential drug-like compounds from Marine Natural Products (MNP) library using the structure-based pharmacophore model.

The validated model *Pharmacophore_01* mapped 3019 compounds from the Marine Natural Products (MNP) library of 14,492 compounds. Subsequent filtering of mapped compounds by Lipinski’s Rule of Five (Ro5) and absorption, distribution, metabolism, excretion and toxicity (ADMET) properties led to further reducing the amount to 135 compounds. These 135 marine drug-like compounds along with reference inhibitor PP242 were escalated for molecular docking with mTOR crystallographic structure and their interactions with residues Leu2185, Asp2195, Ile2237, Gly2238, Trp2239, Val2240, Thr2245, Met2345, Leu2354, Ile2356, and Asp2357 were scrutinized (Figure 2).

### 2.4. Molecular Docking of Retrieved Marine Drug-Like Compounds with mTOR Kinase

The molecular docking process demarcates on the binding affinity, mode of ligand binding in the target protein pocket and also elucidates the interactions of compounds with essential residues. The performance of GOLD software was evaluated by re-docking the co-crystallized ligand PP242 into mTOR binding pocket, resulting in an acceptable RMSD of 0.71 Å (Figure S1). Docking of screened 135 marine drug-like compounds with mTOR kinase domain was then carried out along with reference inhibitor PP242. A total of four marine compounds demonstrated higher Gold scores than PP242 reference inhibitor. The reference compound displayed a lower Gold score of 63.20 as compared with marine compounds hereafter referred to as MNP1, MNP2, MNP3 and MNP4 exhibiting higher Gold scores of 65.48, 65.41, 64.72 and 63.75, respectively (Table 3). The four compounds also demonstrated interactions with the aforementioned residues of the ATP-binding pocket of mTOR kinase domain. A total of 15 molecules exhibited lower docking scores than Torkinib and, therefore, were not considered for further evaluation. However, it was observed that these 15 marine molecules also target similar residues of the mTOR pocket, as seen for the above four compounds (Table S1). Therefore, these four marine compounds and the reference PP242 inhibitor were taken forward for MD simulations to confirm on their stabilities with the mTOR binding pocket.

### 2.5. Binding Mode and Binding Free Energy Analysis of Identified Marine Compounds by Molecular Dynamics Simulations

**Figure 3:**
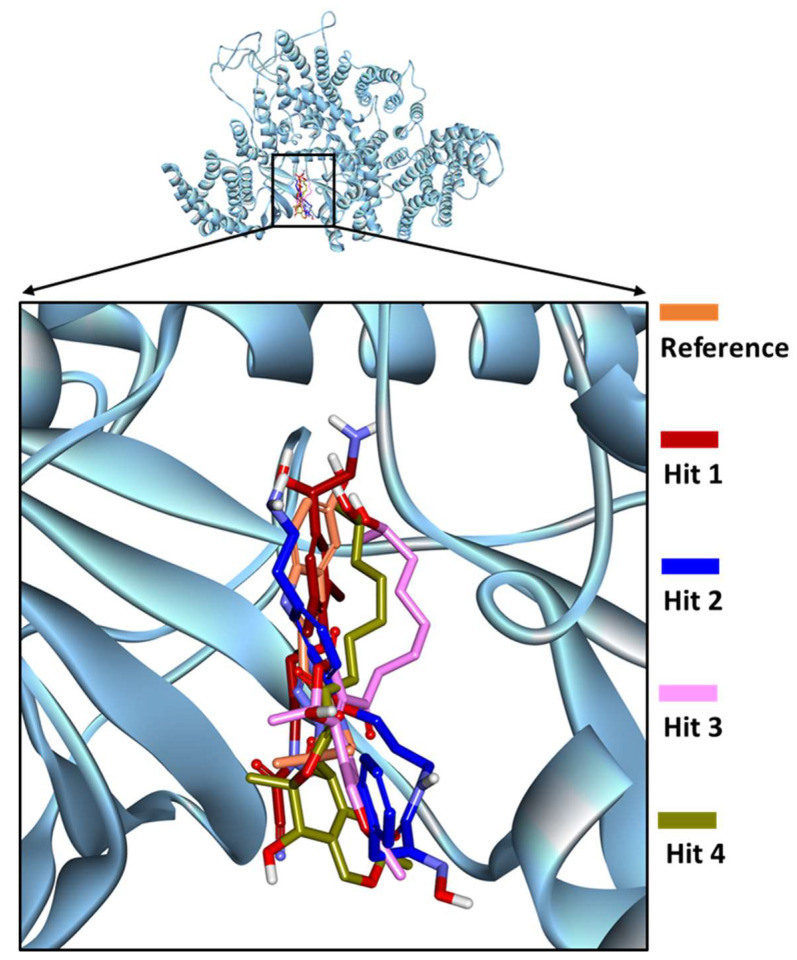
Binding mode of reference PP242 and identified Marine Natural Product (MNP) library hits within the ATP-binding pocket of mTOR kinase domain.

MD simulations employing GROningen Machine for Chemical Simulations (GROMACS) package were executed for the four identified marine compounds docked with the mTOR kinase to comprehend the dynamic behavior at the atomistic level. Simulations were also supplemented with the calculation of binding free energies for the identified hits by MM-PBSA methodology and the compounds were ranked as hits accordingly (Table S2). To gain insight into the binding mode of identified compounds inside the mTOR ATP-binding pocket, the representative structures from the last 5 ns of stable MD trajectories were extracted and subsequently superimposed. Upon scrupulous analysis, it was observed that the identified compounds exhibited a similar binding mode as the PP242 co-crystallized ligand of mTOR (Figure 3).

### 2.6. Characteristic Binding Interaction and Binding Free Energy Analysis of the Confirmed Marine Hits with mTOR ATP-Binding Pocket Residues

#### 2.6.1. mTOR-Hit1 Interaction

**Figure 4:**
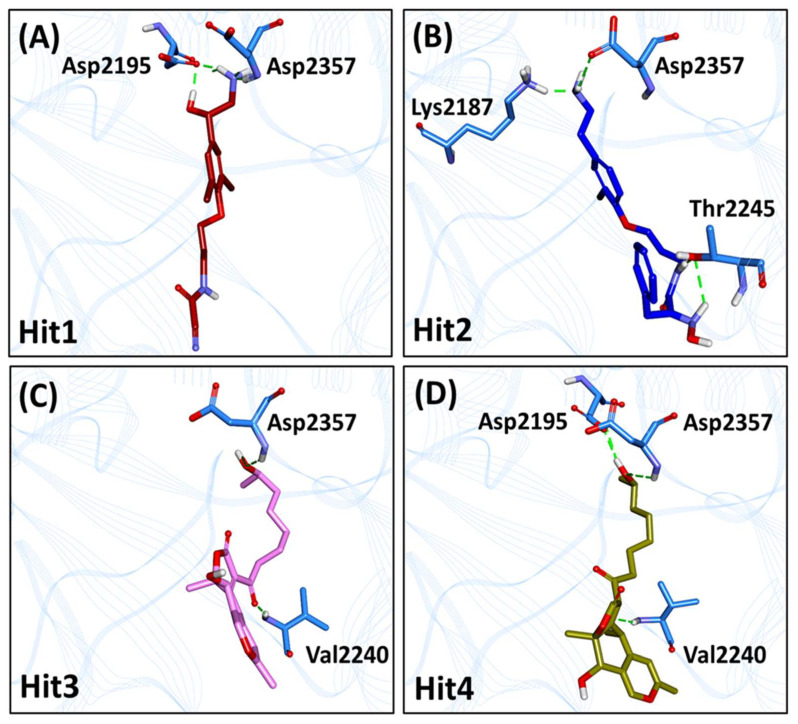
Interactions of (**A**) Hit1, (**B**) Hit2, (**C**) Hit3 and (**D**) Hit4 from Marine Natural Product (MNP) library with key residues of mTOR ATP-binding pocket via hydrogen bonds. The compounds and interacting residues are represented as sticks while the hydrogen bonding interactions are shown as green dashed lines.

**Figure 5:**
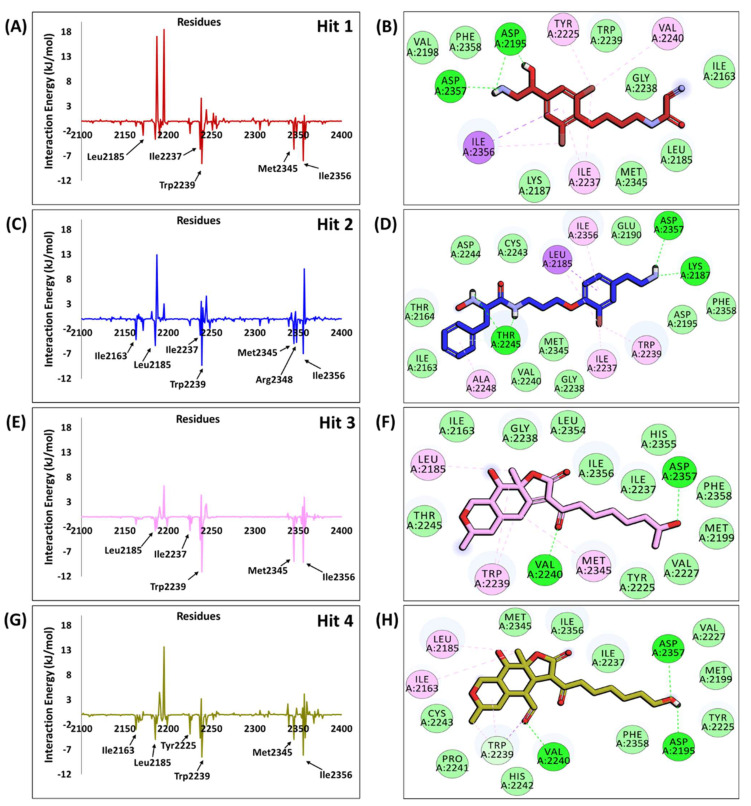
Energy decomposition of individual residues from MM-PBSA contributing to total binding free energy (BFE) for (**A**) Hit1, (**C**) Hit2, (**E**) Hit3 and (**G**) Hit4, and 2D interaction of (**B**) Hit1, (**D**) Hit2, (**F**) Hit3 and (H) Hit4 with residues of mTOR ATP-binding pocket. The hydrogen bonding interactions are shown as green dashed lines, the hydrophobic interactions are shown as pink and purple spheres and the van der Waals interactions are displayed as light green spheres.

The marine compound MNP2 acquired from the docking analysis (Table 3) exhibited the highest BFE of −101.187 ± 17.842 kJ/mol, as investigated by MM-PBSA calculations and, therefore, referred to as Hit1 (Table S2). The estimated BFE gives insight on the diverse components of interaction energy contributing to Hit1 binding. Both electrostatic and van der Waals components contribute a major role in the binding of Hit1 with the ATP-binding pocket of mTOR, where the van der Waals contribution (−151.961 ± 11.779 kJ/mol) is higher than the electrostatic component (−101.485 ± 16.574 kJ/mol). The solvent accessible surface area (SASA) provides a slightly favorable contribution towards the binding of Hit1 with mTOR (−17.534 ± 0.909 kJ/mol). Energy decomposition analysis led to the identification of vital residues contributing to the binding of Hit1 with mTOR (Figure 4A and Figure 5A, Table S2). It was observed that the major contribution for Hit1 binding was from van der Waals interaction with residue Trp2239 (−8.5 kJ/mol) and hydrophobic interaction with residue Ile2356 (−7.9 kJ/mol) which was consistent with its binding mode (Figure 5B). The binding of Hit1 in the mTOR ATP-binding pocket is rendered by hydrogen bond interactions (Figure 4A and Figure 5B) with Asp2195 (bond length: 1.72 Å) and Asp2357 (bond length: 1.96 Å). Additionally, the benzene ring of Hit1 interacted with key residues Tyr2225 (bond length: 4.25 Å), Ile2237 (bond length: 4.70 Å), Val2240 (bond length: 4.94 Å) and Ile2356 (bond length: 4.47 Å) via π-alkyl hydrophobic interactions. Moreover, mTOR residues Ile2163, Leu2185, Lys2187, Val2198, Gly2238, Trp2239, Met2345 and Phe2358 form van der Waals interactions with Hit1 (Figure 5B).

#### 2.6.2. mTOR-Hit2 Interaction

The marine compound MNP3 (hereafter referred to as Hit2) attained from docking analysis (Table 3), demonstrated with BFE of −101.041 ± 20.457 kJ/mol, was observed to be comparable with the BFE of Hit1 (Table S2). Similar to Hit1, contribution of van der Waals (−165.824 ± 15.257 kJ/mol) and electrostatic (−70.466 ± 12.892 kJ/mol) components for the binding of Hit2 with mTOR played a major role than the corresponding SASA energy. The contribution of SASA energy component (−21.107 ± 0.998 kJ/mol) for Hit2 binding was observed to be higher than that of Hit1 with mTOR ATP-binding pocket. The major residues contributing to Hit2 binding with mTOR were also observed to be similar to Hit1 contributing residues (Figure 5C), with Trp2239 (−9.3 kJ/mol) and Ile2356 (−7.0 kJ/mol) forming hydrophobic bonds with Hit2 (Figure 5D). Elucidating on the binding interaction of Hit2 predicted by MD analysis, it was noticed that Hit2 formed three hydrogen bonds (Figure 4B and Figure 5D) with residues Lys2187 (bond length: 1.75 Å), Thr2245 (bond length: 1.80 Å) and Asp2357 (bond length: 2.45 Å). Moreover, the bromo-phenoxy group interacts with residues Leu2185 (bond length: 4.50 Å), Ile2237 (bond length: 3.77 Å) and Trp2239 (bond length: 5.45 Å) via alkyl and π-alkyl hydrophobic interactions. Additional interactions with residues Ala2248 (bond length: 5.18 Å) and Ile2356 (bond length: 4.37 Å) also hold Hit2 in the mTOR hydrophobic pocket via π-alkyl bonds. Furthermore, the residues Ile2163, Thr2164, Glu2190, Asp2195, Gly2238, Val2240, Cys2243, Asp2244, Met2345 and Phe2358 assist in holding Hit2 in the mTOR binding pocket firmly via van der Waals interactions (Figure 5D).

#### 2.6.3. mTOR-Hit3 Interaction

The marine compound MNP1, exhibiting the highest dock score of 65.48 (Table 3) presented with the BFE of −91.924 ± 12.264 kJ/mol (Table S2) and, therefore, ranked and referred as Hit3. It was observed that the van der Waals component was responsible for majorly contributing (−171.314 ± 11.172 kJ/mol) to Hit3 binding with mTOR than electrostatic (−17.958 ± 10.086 kJ/mol) and SASA (−19.290 ± 1.055 kJ/mol) energy components. The van der Waals contribution was observed to be the highest for Hit3-mTOR interaction than mTOR interactions with other hits (Table S2). Additionally, the contribution of this component is 10-fold higher than electrostatic component (−17.958 ± 10.086 kJ/mol) for Hit3-mTOR binding. Emphasizing the major residues contributing to the binding energy of Hit3 with mTOR, van der Waals interaction via Ile2356 (-9.2 kJ/mol) and hydrophobic interactions via Trp2239 (−11.1 kJ/mol) and Met2345 (8.9 kJ/mol) were seen to have a greater impact on binding (Figure 5E,F). Hydrogen bond analysis of the predicted binding mode demonstrated with Hit3 forming two bonds (Figure 4C and Figure 5F) with residues Val2240 (bond length: 2.05 Å) and Asp2357 (bond length: 2.37 Å). Moreover, Hit3 interacts with Leu2185 (bond length: 5.0 Å) from the inner hydrophobic pocket and with Met2345 (bond length: 5.13 Å) of mTOR hydrophobic chamber via alkyl interactions. Two π-alkyl bonds are formed with residue Trp2239 (bond length: 4.32 Å and 4.90 Å) of the hydrophobic chamber. Besides above interactions, van der Waals bonds with residues Ile2163, Met2199, Tyr2225, Val2227, Ile2237, Gly2238, Thr2245, Leu2354, His2355, Ile2356 and Phe2358 contribute to the BFE obtained for Hit3 (Figure 5F).

#### 2.6.4. mTOR-Hit4 Interaction

The marine compound MNP4 (hereafter referred to as Hit4) with lowest dock score among obtained hits (Table 3) demonstrated a BFE of −86.049 ± 14.961 kJ/mol (Table S2). Similar to other hit compounds, the van der Waals component of BFE also contributed greatly to Hit4-mTOR binding (−169.882 ± 11.839 kJ/mol). Correspondingly, the contribution of SASA energy component for Hit4 binding (−19.569 ± 0.780 kJ/mol) was in comparable range with SASA energy for Hit3 binding (−19.290 ± 1.055 kJ/mol). The interaction energy of individual residues towards Hit4-mTOR binding revealed Trp2239 (−8.5 kJ/mol) and Ile2356 (−8.1 kJ/mol) as the major contributing residues via hydrophobic and van der Waals interactions, respectively, as also observed in the above hits (Figure 5G,H). The binding interaction of Hit4 with mTOR revealed that Hit4 interacts with Asp2195 (bond length: 2.03 Å), Val2240 (bond length: 2.40 Å) and Asp2357 (bond length: 2.83 Å) via hydrogen bonds (Figure 4D and Figure 5H). Similar interactions with Asp2195 and Val2240 via hydrogen bonds were also observed in reference structure bonding with mTOR kinase (Figure S2). Elucidating on the hydrophobic interactions of Hit4 with mTOR kinase domain residues, it was observed that Hit4 interacts with hydrophobic chamber residue Ile2163 (bond length: 4.69 Å) and inner hydrophobic pocket residue Leu2185 (bond length: 4.52 Å) via alkyl bonds. Moreover, three π-alkyl bonds (bond lengths: 4.82 Å, 5.22 Å and 5.24 Å) and one π-sigma bond (bond length: 3.73 Å) with hydrophobic chamber residue Trp2239 also contribute to Hit4-mTOR interaction. Furthermore, residues Met2199, Tyr2225, Val2227, Ile2237, Pro2241, His2242, Cys2243, Met2345, Ile2356 and Phe2358 also participate in positioning Hit4 firmly in mTOR binding pocket via van der Waals interactions (Figure 5H).

### 2.7. Evaluation of Drug-Likeness, ADME and Toxicity Properties of Identified mTOR Hits

Knowledge of the drug-likeness and ADME properties of final hits is vital, prior to their consideration as a lead candidate in drug development and/or their usage as anti-cancer drugs. These properties for the final hits were assessed using DS and tabulated. Furthermore, the *Toxicity Prediction (TOPKAT)* module in DS was used to evaluate the toxicity properties of identified hits. Given the structural information of a compound as a query, TOPKAT relies on the concept of Quantitative Structure–Toxicity Relationship (QSTR) models for computing toxicity properties, including AMES mutagenicity and rodent carcinogenicity, based on the National Toxicology Program (NTP) dataset. According to the U.S. NTP protocol, a compound’s carcinogenicity is assessed by testing it in female and male sexes of mouse as well as rat. As per the calculations, all hits were observed to obey the Lipinski’s Ro5 and displayed molecular weight of less than 500 Da with predicted octanol/water partition coefficient logP of less than 5.0 and estimated hydrogen bond donors and acceptors of less than 5 and 10, respectively (Table S2). The hits also followed the Veber’s rule except for Hit2 with 10 rotatable bonds. Additionally, the identified hits satisfied the ADME properties demonstrating low blood–brain barrier (BBB) penetration, no inhibition of CYP2D6, good human intestinal absorption (HIA) as well as good aqueous solubility (Table S3). The results from TOPKAT analysis suggested that our identified hits demonstrated non-carcinogenicity in both sexes of rat models, while Hit2 appeared to be carcinogenic in mouse male (Table S4). According to the TOPKAT AMES mutagenicity analysis, Hit2 was displayed as being a mutagen while other hits were observed to be non-mutagenic (Table S4). 

### 2.8. Novelty and Source Documentation of Identified mTOR Inhibitors

As a final assessment, the source of the identified marine hits was evaluated. For this purpose, the PubChem chemistry database (https://pubchem.ncbi.nlm.nih.gov/, (accessed on 11 February 2021)) was searched with Chemical Abstracts Service (CAS) numbers/MNP IDs of the respective hits as queries to identify if our hits have already been reported in the literature for mTOR kinase or other target proteins. From PubChem literature analysis, it was found that the compound Hit1 (7-Hydroxyceratinamine) is a cyanoformamide-containing metabolite originating from a Micronesian sponge, *Aplysinella* sp [36]. In addition, Hit2 was found to be a marine alkaloid isolated from the sponge *Psammaplysilla purpurea* [37]. The source for Hit3 could not be identified while Hit4 was recognized as a phytolide of the *Colletotrichum boninense* fungal origin [38]. As per the literature analysis, the four identified hits have not been reported as inhibitors of mTOR kinase. Therefore, the identified hits in the present study provide valuable alternatives as therapeutic candidates for further lead optimization.

## 3. Discussion

Kinase proteins act as chief regulatory entities in cellular biology. Moreover, their hyperactivation leads to several pathologies, including cancer. Therefore, kinases have become essential pharmacological targets and the discovery of small molecule drugs is a predominant scientific activity to mitigate the cancerous ailments. The mammalian target of rapamycin, mTOR is a dual specificity protein kinase which phosphorylates serine/threonine as well as tyrosine residues. The deregulation of mTOR is associated with diabetes, obesity, aging and various types of cancer [39,40,41,42,43]. The PI3K/AKT/mTOR pathway has been largely implicated in the tumorigenesis and progression of aforementioned cancers. Specific mTOR inhibitors are currently in various stages of clinical trials [44]. The mTOR inhibitors appear to be well tolerated combined with adverse effects, including myelosuppression, metabolic abnormalities, stomatitis and skin reactions, among other abnormalities [45]. These inhibitors can be classified into first generation allosteric inhibitors such as rapamycin and its analogues [46] and second generation ATP-competitive inhibitors. Several ATP-competitive mTOR inhibitors have been discovered and being tested in clinical trials including selective inhibitors like CC-223, INK-128 and OSI-027 [47]. Unlike the former category of inhibitors, the ATP-competitive mTOR inhibitors block the ATP-binding catalytic site as well as reduce the activity of mTORC1 and mTORC2 complexes and hence has become an effective strategy to suppress both the ATP and allosteric sites. Encouraged from these efforts, we pursued our research strategy to identify natural product compounds as mTOR ATP-competitive inhibitors by applying structure-based pharmacophore modelling. Such a pharmacophore strategy works towards exploiting the key interactions between the protein residues and the bound co-crystallized ligand [48].

Marine extracts have displayed a great potential as an essential source of new drugs. Marine environments remain extensively unexplored despite being a huge source of bioactive compounds against cancer. Aquatic habitats have produced a variety of marine-derived alkaloids, triterpenoids and peptides. Intriguingly, a purple sponge extract of Turkish marine origin was shown to display promising activity against a panel of tyrosine kinases and cell lines including A549, A375, KMS-12PE and K562 cancer cell lines [32]. Similarly, the brown algae-derived polysaccharide, Fucoidan, was shown to exert anti-cancer effects not only through cell cycle arrest but also by indirectly killing cancerous cells by activation of natural killer cells and macrophages. Fucoidan was also shown to demonstrate inhibitory activity against cancer A549, MCF-7, PC-3 and SMMC-7721 cells [31]. In addition, Fascaplysin which is a bis-indole of a marine sponge demonstrated anticancer activity as CDK4 inhibitor in lung cancer cell line [46]. The antitumor potential of marine algae-derived compounds has also been extensively reviewed recently [24]. We therefore designed our study to target the mTOR dysregulation in cancer using marine-derived bioactive natural products employing a series of computational methods. Using a structure-based pharmacophore approach, we have developed a pharmacophore model from mTOR protein structure co-crystallized with ligand PP242 (Figure 1). Torkinib/PP242 is a selective ATP-competitive inhibitor of mTOR with promising anti-cancer activity over numerous cancer types [49]. A total of 14,492 compounds of marine origin were screened with the pharmacophore model as a query, deriving 3019 compounds as candidates mapping the pharmacophore model. Subsequently, a drug-like database was prepared employing Lipinski’s Ro5 and ADMET rules, thereby retrieving 135 compounds (Figure 2). Molecular docking-based interaction screening of these 135 compounds resulted in the identification of four compounds with higher docking scores than PP242 and similar interactions with the ATP-binding pocket of mTOR (Table 3). Escalating the identified four compounds to molecular dynamics simulations for observing their behavior at the atomistic level gave insights into the critical residues required for the specific mTOR inhibition.

**Figure 6:**
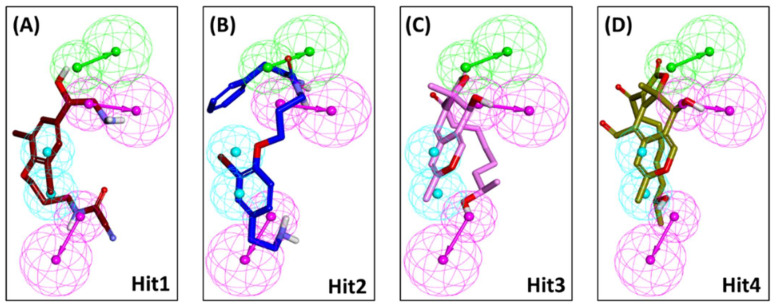
Alignment of the (**A**) Hit1, (**B**) Hit2, (**C**) Hit3 and (**D**) Hit4 with the pharmacophoric features. All hits represent the HBA (hydrogen bond acceptor), HBD (hydrogen bond donor) and Hy (hydrophobic) features of *Pharmacophore_01*.

The structure-based pharmacophore and MD analysis of the mTOR-PP242 crystal structure revealed that the inhibitor targets key residues Asp2195, Gly2238 and Val2240 via hydrogen bonds entailing essential pharmacophoric features including HBD and HBA (Figure 1B and Figure S2A). It has been elucidated in previous studies that hydrogen bonds with Asp2195 and Val2240 are indispensable for mTOR inhibitory activity [50,51,52,53,54,55,56]. In the current study, Hit1 was observed to retain the hydrogen bonding interaction with Asp2195 (Table 3, Figure 4A), while Hit4 interaction with both residues was preserved even after 30 ns of production simulation run (Table 3, Figure 4D). Additionally, our hits formed hydrogen bonds with catalytic hydrophilic residue Asp2357 of the mTOR ATP-binding site which offers a level of specificity for our hits towards mTOR than PI3K (Figure 4) [51,52,53,54,55]. The compound Hit2 also formed additional hydrogen bonds with residues Lys2187 and Thr2245 (Figure 4B) and interactions with these residues were also reported in previously published studies [22,51,52,55]. A recent study reported natural products as mTOR ATP-binding site and rapamycin binding site inhibitors derived from three databases—Marine Natural Products Library, SuperNatural II and ZINC natural products—and also provided experimental evidence against mTOR for eleven compounds [23]. However, the marine compounds identified from their studies as mTOR inhibitors are distinct from our proposed marine natural product hits. In addition to the aforementioned hydrogen bonds, our hits are also characterized by several hydrophobic and van der Waals interactions (Figure 5). In particular, interaction with residue Trp2239 of the hydrophobic chamber was observed via π-stacking bonds or van der Waals interaction for our hits, similar to Torkinib interaction with mTOR (Figure 5 and Figure S2B). Earlier studies reported that Trp2239 is not present in canonical protein kinases such as PI3K and interaction with this residue provides for mTOR inhibitor specificity over PI3K [56,57]. All of these results suggest that our hits may be selective mTOR inhibitors. From our per-residue contribution analysis, Trp2239 was observed to contribute the highest for Hit3 binding (−11.1 kJ/mol) (Figure 5E) followed by Hit2 (−9.3 kJ/mol) (Figure 5C), Hit1 (−8.53 kJ/mol) (Figure 5A) and Hit4 (−8.52 kJ/mol) (Figure 5G) binding with mTOR. The Trp2239 contributed the highest energy for binding of Hit3 among the four identified hits forming strong interactions via π hydrophobic bonds as observed (Figure 5F), thus suggesting a higher level of selectivity of Hit3 binding with mTOR. In a recently published review, the significant residues involved in ligand selectivity towards mTOR over PI3K were reviewed in detail and reported as Arg770, Glu2190 and Cys2243 [50]. Our identified Hit2 displayed bonds with Glu2190 and Cys2243 via van der Waals interactions (Figure 5D), while Cys2243 was also observed to support Hit4 via van der Waals interactions in the ATP-binding pocket of mTOR (Figure 5H). The above-mentioned analyses indicate that our identified marine hits have better binding affinity, as computed from MM-PBSA binding free energy scores, and also seem to confer comparable selectivity towards mTOR as the previously identified selective inhibitor, Torkinib. Correspondingly, the identified hits also portray the essential pharmacophoric features required for mTOR inhibition, similar to Torkinib (Figure 1 and Figure 6). 

As a final analysis, the four identified marine hits were scrutinized for their physicochemical, pharmacokinetic and toxicity properties to evaluate their in vivo disposition prior to their consideration as therapeutic anti-cancer drugs. The drug-likeness properties and ADME results demonstrated that the identified hits satisfied the Ro5 criteria, could be absorbed easily in the human intestine, show good aqueous solubility, do not inhibit the CYP2D6 enzyme and do not penetrate the BBB (Table S3). Despite presenting with good Ro5 properties, Hit2 displayed with the upper limit value (i.e., 10) in the case of Veber’s rule of rotatable bonds. In addition, the hits were identified as non-carcinogenic in rodent NTP models and non-AMES mutagenic, except for Hit2, which showed carcinogenicity in the mouse male NTP model and also showed AMES mutagenicity (Table S4). Although Hit2 exhibited with a BFE of −101.041 kJ/mol (Table S2) and presented with key intermolecular interactions with mTOR (Figure 4B and Figure 5D), it cannot be considered as a therapeutic candidate for further drug optimization, owing to its toxicity properties. Overall, we believe the potentiality of Hit1, Hit3 and Hit4 as alternatives and their scaffolds can be further explored for developing efficient ATP-binding site mTOR inhibitors for cancer therapeutics. Though the in vitro studies of our identified marine hits are further required to clinically substantiate these findings, structure-based pharmacophore modeling and virtual screening strategy can be very useful to design potent molecules as mTOR inhibitors in future drug discovery studies. In addition, our study characterizes a platform for future discovery of novel natural chemotherapeutic drugs from marine natural habitat.

## 4. Materials and Methods

### 4.1. Structure-Based Pharmacophore Model Generation

Receptor-based pharmacophore model delves into the catalytic site of the target protein bound with its inhibitor to identify essential pharmacophoric features effective for inhibition [58]. The structure of mTOR target protein bound with its ATP-site inhibitor PP242 (PDB ID: 4JT5, 3.45 Å) was retrieved from Research Collaboratory for Structural Bioinformatics (RCSB) Protein Data Bank (PDB) and considered for the generation of a structure-based pharmacophore model [56]. PP242 (also known as Torkinib) is a potent and specific inhibitor of mTOR kinase domain proven to be more effective than traditional mTOR inhibitor rapamycin [14,59]. Subsequently, the residues within 9 Å around PP242 were considered and *Receptor-Ligand Pharmacophore Generation* module embedded in Discovery Studio (DS) v.2018 was employed for model generation. The model with the highest selectivity score was chosen for subsequent validation.

### 4.2. Validation of Generated Pharmacophore Model

Given a particular dataset, pharmacophore validation is a quintessential criterion for ensuring efficient retrieval of active target protein compounds. Accordingly, the model chosen from the above-mentioned criteria was validated by Güner–Henry approach (decoy set) approach [60] for evaluating the robustness of the pharmacophore model on the basis of goodness of fit (GF) score in the range of 0 (null model) and 1 (ideal model) [58,61,62].

$$GF=\;\left(\frac{Ha}{4HtA}\right)\left(3A+Ht\right)\;\times \;\left\{1-\frac{Ht-Ha}{D-A}\right\}$$

The decoy set approach was instigated by evaluating the selected pharmacophore model on an external dataset (D) of 300 compounds obtained from the same biological assay [63]. This dataset was divided into 50 compounds exhibiting IC_50_ < 100 nmol/L, referred to as active (A) mTOR inhibitors, with the remaining compounds being inactive. The *Ligand Pharmacophore Mapping* was executed for dataset screening, complemented with *FAST* algorithm and the GF score was calculated.

### 4.3. Virtual Screening of Marine Natural Product Library

The validated pharmacophore model was escalated to screen the Marine Natural Product (MNP) library composed of 14,492 compounds (http://docking.umh.es/downloaddb, (accessed on 11 December 2020). The DS module *Ligand Pharmacophore Mapping* was employed in pursuit of identifying scaffolds mapping the pharmacophoric features. The compounds obtained from screening were further subjected to filtering by Lipinski’s Rule of Five (Ro5) [64], Veber’s rules [65] and pharmacokinetics by absorption, distribution, metabolism, excretion and toxicity (ADMET) for retrieval of drug-like compounds. Accordingly, the *Filter by Lipinski and Veber Rules* and *ADMET Descriptors* modules within DS were recruited for evaluation. The Ro5 and Veber’s rules collectively oversee the physiochemical properties for efficient retrieval of compounds with molecular weight ≤500 kDa, number of hydrogen bond donors ≤5, compound’s lipophilicity (log*P*) ≤5, number of hydrogen bond acceptors ≤10 and the number of rotatable bonds ≤10. The drug-like compounds so obtained were subjected to molecular docking with the mTOR kinase domain along with PP242 as reference inhibitor.

### 4.4. Molecular Docking of Drug-Like Compounds with mTOR Kinase Domain

Molecular docking methods explore the binding conformations adopted within the catalytic sites of macromolecular protein targets, thereby evaluating the vital phenomena for the intermolecular recognition process [66]. The drug-like compounds obtained from the above filtering criterion were subjected to molecular docking in Genetic Optimisation for Ligand Docking (GOLD) v5.2.2 automated docking software [67]. The compounds were evaluated on the basis of the GOLD default scoring function—Gold score [68]. This fitness score functions by scoring the summation of protein-ligand van der Waals interaction energy and hydrogen-bonding energy. The retrieved 3D crystallographic structure of mTOR (PDB ID: 4JT5) complexed with PP242 ATP-competitive inhibitor was prepared by utilizing the *Clean Protein* module in DS. Protein preparation was further carried out by adding the missing residues and hydrogen atoms. The water molecules were removed along with the bound PP242 ligand. Prior to docking, the performance of GOLD docking was assessed by re-docking the native PP242 ligand into mTOR. The drug-like compounds were subjected to minimization employing *Minimize Ligands* module in DS, preceding docking. Subsequently, molecular docking of drug-like compounds with mTOR was followed by applying the same docking parameters utilized for PP242 docking allowing for generation of 50 conformers per ligand. Clustering of the obtained conformations was carried out to obtain the largest cluster and each compound in the cluster was examined on the basis of higher Gold score than reference compound PP242, binding mode within the mTOR catalytic site and molecular interactions with the key residues of the mTOR kinase domain. The selected potential compounds acquired from this strategy were refined by MD simulations.

### 4.5. Molecular Dynamics Simulation of Identified Hits

MD simulation studies of compounds identified from above docking were extensively carried out to decipher the molecular dynamics in water and comprehend the interaction of hit compounds with vital residues of mTOR active site at the atomistic level. The docked structures of these marine hits in complex with mTOR were used as initial coordinates for simulations with GROMACS v2018 [69]. The mTOR and hit compounds were applied with CHARMm27 force field [70] and topologies generated with SwissParam [71] fast force field generation tool, respectively. The dodecahedron water box was utilized to solvate the systems with TIP3P water model and further neutralized with Na^+^ counter ions. The steepest descent energy minimization was performed to dodge bad contacts and this was followed by a two-fold equilibration. The NVT (constant number of particles, volume and temperature) equilibration at 300 K with a V-rescale thermostat supplemented with NPT (constant number of particles, pressure and temperature) equilibration at 1 bar pressure with a Parrinello-Rahman barostat [72] was orchestrated, each for 1000 ps. The LINear Constraint Solver (LINCS) [73] and SETTLE algorithms [74] were applied to monitor bond constrains and the geometry of water molecules. The long-range electrostatic interactions were calculated by means of Particle Mesh Ewald (PME) [75] and the equilibrated systems were subjected to production simulation runs of 30 ns. The acquired MD results were visualized and interpreted manually in visual molecular dynamics (VMD) [76] and DS. The binding free energy (BFE) scores were further computed for hit compounds by MM-PBSA executing *g_mmpbsa* tool implemented in GROMACS [77]. For this purpose, 40 frames of mTOR-ligand complexes were selected evenly from the last 10 ns of MD trajectories and the BFE *∆G_bind_* was computed as per the below equation.

$$∆G_{bind}=\;G_{complex}-\left(G_{protein}+\;G_{ligand}\right)$$

## 5. Conclusions

A structure-based pharmacophore model, exploiting the crystal structure of mTOR serine/threonine kinase with its bound selective inhibitor Torkinib revealed fundamental pharmacophoric features required for mTOR inhibition at its ATP-binding pocket. A systematic virtual screening strategy with the model as a query, retrieved 3019 compounds from the Marine Natural Products library and subsequent filtering via Lipinski’s Ro5, Veber’s rule, and ADMET was able to acquire 135 drug-like compounds. Molecular docking of these compounds at the ATP-binding site of mTOR procured four marine compounds with higher dock scores than Torkinib and significant binding interactions with key residues of the pocket. These compounds also presented with good binding free energy scores and the energy contribution of essential residues unveiled that our hits can inhibit mTOR via Glu2190, Trp2239 and Cys2243 deemed requisite for selective inhibition over PI3K. The in silico ADME and toxicity analysis suggests three out of the four identified hits with acceptable pharmacokinetic profile for their in vivo disposition. Additionally, the biological origin of these hits was identified as marine fungus and sponge. Overall, we believe that our hits provide scaffolds for future drug optimization studies and, therefore, recommend these hit compounds from marine natural habitat as therapeutics for the treatment of cancer. The identification of marine-derived natural compounds embodies an essential platform for future drug discovery studies against various protein targets implicated in cancers.

## Figures and Tables

**Table 1:** Structure-based pharmacophore models with their generated features.

Pharmacophore Models	Number of Features	Feature Set *	Selectivity Score
*Pharmacophore_01*	5	ADDHH	9.2973
*Pharmacophore_02*	4	DDHH	7.7825
*Pharmacophore_03*	4	ADDH	7.7825
*Pharmacophore_04*	4	ADDH	7.7825
*Pharmacophore_05*	4	ADHH	6.8689
*Pharmacophore_06*	4	ADHH	6.8689

* A: hydrogen bond acceptor (HBA); D: hydrogen bond donor (HBD); H: hydrophobic (Hy).

**Table 2:** Decoy set validation of *Pharmacophore_01* from an external database composed of active and inactive mTOR inhibitors.

S. No.	Parameters	Values
1	Total number of compounds in the database (D)	300
2	Total number of active compounds in the database (A)	50
3	Total number of hits retrieved by pharmacophore model from the database (Ht)	61
4	Total number of active compounds in the hit list (Ha)	49
5	% Yield of active ((Ha/Ht) × 100)	80
6	% Ratio of actives ((Ha/A) × 100)	98
7	False negatives (A-Ha)	1
8	False positives (Ht-Ha)	12
9	Goodness of fit score (GF)	0.80

**Table 3:** The docking scores and intermolecular interactions of reference PP242 and Marine Natural Product (MNP) library compounds with mTOR kinase domain (PDB ID: 4JT5).

Compound No.	MNP ID (CAS No *)	Gold Score	Hydrogen Bond Interactions	Hydrophobic and van der Waals Interactions
1 (MNP1)	200936-85-2	65.48	Val2240, Asp2357	Leu2185, Lys2187, Leu2192, Asp2195, Tyr2225, Val2227, Ile2237, Gly2238, Trp2239, His2242, Cys2243, Asp2244, Thr2245, Met2345, Arg2348, Ile2356, Phe2358
2 (MNP2)	230295-94-0	65.41	Asp2195, Trp2239, Val2240	Leu2185, Lys2187, Leu2192, Met2199, Tyr2225, Val2227, Ile2237, Pro2241, His2242, Cys2243, Met2345, Arg2348, Ile2356, Asp2357, Phe2358
3 (MNP3)	149636-93-1	64.72	Trp2239, Val2240	Leu2185, Lys2187, Glu2190, Leu2192, Asp2195, Tyr2225, Ile2237, Trp2239, Val2240, Pro2241, His2242, Cys2243, Met2345, Arg2348, Ile2356, Asp2357, Phe2358
4 (MNP4)	200936-84-1	63.75	Asp2195, Val2240, Asp2357	Ile2163, Leu2185, Leu2192, Met2199, Tyr2225, Val2227, Ile2237, Trp2239, Pro2241, His2242, Cys2243, Asp2244, Thr2245, Met2345, Arg2348, Ile2356
5 (PP242)	Reference (1092351-67-1)	63.20	Asp2195, Gly2238, Val2240	Ile2163, Leu2185, Lys2187, Met2199, Tyr2225, Ile2237, Trp2239, Cys2243, Thr2245, Met2345, Ile2356, Asp2357, Phe2358

* CAS: Chemical Abstracts Service.

## Data Availability

Data are contained within the article.

## Supplementary Materials

The following are available online at https://www.mdpi.com/1424-8247/14/3/282/s1, Figure S1: Overlay of the docked pose (orange) PP242 inhibitor of mTOR kinase with its crystal structure conformation (green) in PDB ID: 4JT5. Figure S2. (A) 3D and (B) representation of interaction between PP242 and catalytic residues of the ATP-binding pocket of mTOR. The compounds and interacting residues are represented as sticks. The hydrogen bonding interactions are shown as green dashed lines, the hydrophobic interactions are shown as pink and purple spheres and the van der Waals interactions are displayed as light green spheres. Table S1. The docking scores and intermolecular interactions of reference PP242 and Marine Natural Product (MNP) library compounds with mTOR kinase domain (PDB ID: 4JT5). Table S2. Binding free energy scores of identified Marine Natural Product (MNP) library hits with mTOR calculated through MM-PBSA methodology. Table S3. Lipinski’s and ADME properties of identified marine hits to determine their drug-likeness. Table S4. Toxicity properties of identified marine hits to determine their drug likeness.
