# Supplementary material for: Marine-Derived Natural Products as ATP-Competitive mTOR Kinase Inhibitors for Cancer Therapeutics
Source: Pharmaceuticals (Basel). 2021 Mar 21;14(3):282. doi: 10.3390/ph14030282 (PMC8003863; doi:10.3390/ph14030282)
Supplement: Supplementary file 1 [file pharmaceuticals-14-00282-s001.pdf]

pharmaceuticals MDPI

Supplementary Material:

Marine-derived Natural Products as ATP-competitive mTOR Kinase Inhibitors for Cancer Therapeutics

Shraddha Parate[1], Vikas Kumar[2], Gihwan Lee[1], Shailima Rampogu[2], Jong Chan Hong[1,*] and Keun Woo Lee[2,*]

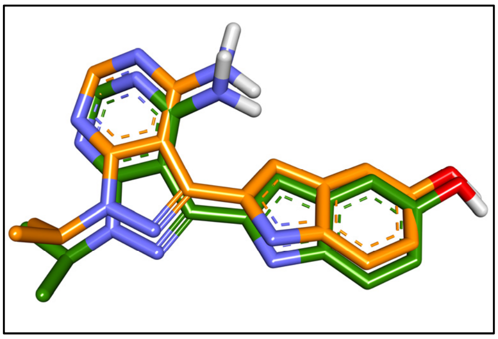

Figure S1. Overlay of the docked pose (orange) PP242 inhibitor of mTOR kinase with its crystal structure conformation (green) in PDB ID: 4JT5.

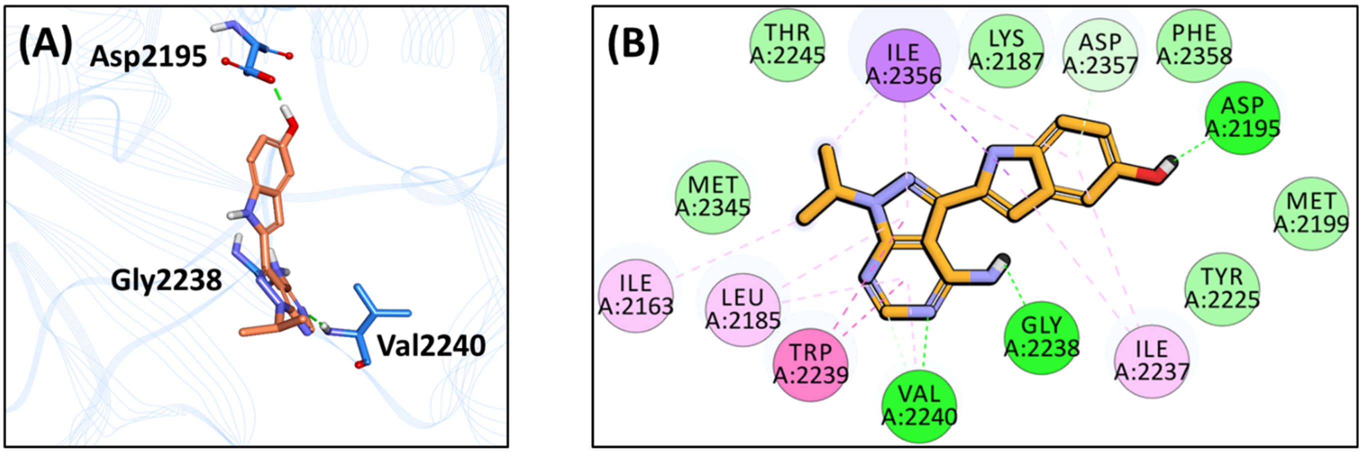

Figure S2. (A) 3D and (B) representation of interaction between PP242 and catalytic residues of the ATP-binding pocket of mTOR. The compounds and interacting residues are represented as sticks. The hydrogen bonding interactions are shown as green dashed lines, the hydrophobic interactions are shown as pink and purple spheres and the van der Waals interactions are displayed as light green spheres.

Table S1. The docking scores and intermolecular interactions of reference PP242 and Marine Natural Product (MNP) library compounds with mTOR kinase domain (PDB ID: 4JT5).

Compound No.	MNP ID (CAS No*)	Gold Score	Hydrogen Bond Interactions	Hydrophobic and van der Waals Interactions
1 (PP242)	Reference (1092351-67-1)	63.20	Asp2195, Gly2238, Val2240	Ile2163, Leu2185, Lys2187, Met2199, Tyr2225, Ile2237, Trp2239, Cys2243, Thr2245, Met2345, Ile2356, Asp2357, Phe2358
2	228113-43-7	62.44	Asp2191, Asp2195	Leu2185, Lys2187, Glu2190, Leu2192, Gln2194, Met2199, Tyr2225, Ile2237, Val2240, Met2345, Ile2356, Phe2358, Gly2359
3	346585-46-4	62.18	Lys2187, Asp2195	Leu2185, Tyr2225, Ile2237, Gly2238, Trp2239, Val2240, Cys2243, Asp2244, Thr2245, Ala2248, Met2345, Arg2348, Ile2356, Asp2357, Phe2358
4	121678-87-3	60.75	Cys2243	Ile2163, Leu2185, Lys2187, Leu2192, Asp2195, Met2199, Tyr2225, Val2227, Ile2237, Trp2239, Val2240, Asp2244, Thr2245, Met2345, Arg2348, Ile2356, Asp2357, Phe2358
5	853885-46-8	60.61	Val2240	Ile2163, Leu2185, Lys2187, Leu2192, Asp2195, Tyr2225, Ile2237, Trp2239, Cys2243, Thr2245, Met2345, Leu2354, Ile2356, Phe2358
6	77136-61-9	59.35	Ser2165, Lys2187	Ile2163, Gln2167, Pro2169, Leu2185, Glu2190, Tyr2225, Ile2237, Gly2238, Trp2239, Val2240, Cys2243, Asn2343, Met2345, Ile2356, Asp2357
7	134029-41-7	57.73	Val2240	Leu2185, Lys2187, Leu2192, Asp2195, Met2199, Tyr2225, Val2227, Gly2238, Ile2237, Trp2239, Pro2241, Cys2243, Met2345, Ile2356, Phe2358
8	121678-86-2	57.68	Asp2195, Asp2357	Ile2163, Leu2185, Lys2187, Leu2192, Met2199, Tyr2225, Val2227, Ile2237, Trp2239, Val2240, Cys2243, Asp2244, Thr2245, Met2345, Leu2354, Ile2356
9	173791-67-8	56.88	Lys2187, Asp2195	Leu2185, Leu2192, Val2240, Met2345, Asp2357, Phe2358
10	77136-63-1	56.82	Gly2238	Ile2163, Leu2185, Lys2187, Leu2192, Asp2195, Met2199, Tyr2225, Val2227, Ile2237, Trp2239, Val2240, Cys2243, Thr2245, Met2345, Ile2356, Asp2357, Phe2358
11	88899-59-6	56.34	Asp2195, Asp2357	Leu2185, Lys2187, Met2199, Tyr2225, Ile2237, Gly2238, Trp2239, Val2240, Cys2243, Met2345, Ile2356, Phe2358
12	77136-62-0	54.06	Asp2195	Ile2163, Leu2185, Lys2187, Leu2192, Val2227, Ile2237, Gly2238, Trp2239, Val2240, Cys2243, Thr2245, Met2345, Ile2356, Asp2357, Phe2358
13	123060-45-7	53.58	Cys2243, Met2345	Ile2163, Leu2185, Ile2237, Gly2238, Val2240, Asp2244, Arg2348, Leu2354, Ile2356
14	851706-23-5	53.57	Lys2187	Ile2163, Leu2185, Asp2195, Ile2237, Trp2239, Val2240, Cys2243, Thr2245, Asp2244, Ser2342, Met2345, Ile2356, Asp2357
15	49644-26-0	53.32	Gly2238	Ile2163, Ser2165, Pro2169, Leu2185, Lys2187, Leu2192, Asp2195, Met2199, Tyr2225, Val2227, Ile2237, Trp2239, Val2240, Cys2243, Thr2245, Met2345, Ile2356, Asp2357, Phe2358
16	604807-25-2	53.06	Ser2165, Lys2187	Ile2163, Gln2167, Pro2169, Leu2185, Glu2190, Trp2239, Val2240, Cys2243, Asp2244, Thr2245, Met2345, Ile2356

*CAS: Chemical Abstracts Service

Table S2. Binding free energy scores of identified Marine Natural Product (MNP) library hits with mTOR calculated through MM-PBSA methodology.

MNP Hits (CAS No.*)	van der Waals (kJ/mol)	Electrostatic (kJ/mol)	Polar solvation (kJ/mol)	SASA energy (kJ/mol)	Binding energy ΔG_{bind} (kJ/mol)
Hit1 (230295-94-0)	-151.961+/-11.779	-101.485+/-16.574	169.793+/-26.091	-17.534+/-0.909	-101.187+/-17.842
Hit2 (149636-93-1)	-165.824+/-15.257	-70.466+/-12.892	156.356+/-20.580	-21.107+/-0.998	-101.041+/-20.457
Hit3 (200936-85-2)	-171.314+/-11.172	-17.958+/-10.086	116.637+/-18.216	-19.290+/-1.055	-91.924+/-12.264
Hit4 (200936-84-1)	-169.882+/-11.839	-29.171+/-9.362	132.573+/-13.743	-19.569+/-0.780	-86.049+/-14.961

*CAS: Chemical Abstracts Service

Table S3. Lipinski's and ADME properties of identified marine hits to determine their drug-likeness.

MNP Hits[a]	MW[b]	HBD[c]	HBA[d]	logP[e]	Rotatable Bonds[f]	HIA[g]	BBB[h]	Aqueous Solubility[i]	CYP2D6 Prediction
Hit1	421.085	4	6	1.185	7	0	3	3	False
Hit2	434.327	4	6	2.886	10	0	3	3	False
Hit3	374.428	2	6	1.675	7	0	3	3	False
Hit4	402.438	2	7	1.287	8	0	3	3	False

[a]MNP: Marine Natural Products, [b]MW: Molecular weight in Dalton, [c]HBD: Estimated number of hydrogen bond donors, [d]HBA: Estimated number of hydrogen bond acceptors, [e]logP: Predicted octanol/water partition co-efficient, [f]Rotatable Bonds: Estimated number of rotatable bonds, [g]HIA: Human intestinal absorption level, [h]BBB: Estimated blood brain barrier level, [i]Solubility: Predicted aqueous solubility.

Table S4. Toxicity properties of identified marine hits to determine their drug-likeness.

MNP Hits[a]	Rat Female Carcinogenicity	Rat Male Carcinogenicity	Mouse Female Carcinogenicity	Mouse Male Carcinogenicity	AMES Mutagenicity[b]
Hit1	Non-carcinogen	Non-carcinogen	Non-carcinogen	Non-carcinogen	Non-mutagen
Hit2	Non-carcinogen	Non-carcinogen	Non-carcinogen	Carcinogen	Mutagen
Hit3	Non-carcinogen	Non-carcinogen	Non-carcinogen	Non-carcinogen	Non-mutagen
Hit4	Non-carcinogen	Non-carcinogen	Non-carcinogen	Non-carcinogen	Non-mutagen

[a]MNP: Marine Natural Products, [b]AMES: Salmonella typhimurium reverse mutation assay
